# Real-world treatment patterns and survival outcomes in men with metastatic castration-resistant prostate cancer in Finland: a national, population-based cohort study

**DOI:** 10.2340/1651-226X.2025.42173

**Published:** 2025-01-29

**Authors:** Antti Rannikko, Olivia Hölsä, Trude Ågesen, Mattias Ekman, Riikka Mattila

**Affiliations:** a Department of Urology, University of Helsinki and Helsinki University Hospital, Helsinki, Finland; b Research Programme in Systems Oncology, Faculty of Medicine, University of Helsinki, Helsinki, Finland; c Medaffcon Oy, Espoo, Finland; d AstraZeneca Nordic, Oslo, Norway; e AstraZeneca Nordic, Stockholm, Sweden

**Keywords:** Real-world evidence, prostate cancer, mCRPC, androgen receptor pathway inhibitors, treatment, overall survival, androgen receptor antagonists

## Abstract

**Background:**

Metastatic castration-resistant prostate cancer (mCRPC) treatment is advancing yet Nordic, real-world evidence for its use is scarce. In this population-based cohort study, we describe characteristics of patients with mCRPC, and their treatment patterns and survival outcomes in Finland.

**Methods:**

Incident patients with mCRPC diagnosed during 2013–2021 were identified from data lakes in two large and representative, Finnish hospital districts, and linked to data on drug purchases and causes of death from national registries.

**Results:**

Of a total of 31,307 patients with prostate cancer, 2,475 progressed to mCRPC during 2013–2021. Those who received no life-prolonging treatment(s) (28% overall) were older with more comorbidities than treated patients. After 2018, the proportion of patients who received life-prolonging treatments increased from 61% to 80%. Of those who received treatment before androgen receptor pathway inhibitors (ARPIs) were reimbursed as first-line (1L) treatment for mCRPC in Finland, 68% received docetaxel, 19% abiraterone and 12% enzalutamide 1L; post-reimbursement, 4% received docetaxel, 24% abiraterone and 71% enzalutamide 1L. Median overall survival for treated patients with mCRPC was 28.3 (95% CI: 26.3–30.4) and 38.5 (95% CI: 32.7–42.1) months pre- and post-reimbursement of 1L-ARPIs, respectively.

**Interpretation:**

The ARPI reimbursement status changes significantly influenced treatment patterns for mCRPC in Finland, favouring enzalutamide over docetaxel. This expanded the pool of men eligible for 1L treatment and improved overall survival by a median of 10 months. These findings highlight the importance of health policy decisions in shaping treatment strategies and patient outcomes in prostate cancer.

## Introduction

The most common cancer and second most common cause of cancer death for men in many Western countries, including Finland [1], is prostate cancer (PCa). While survival of patients with PCa is generally good [2], 10–20% of men will eventually develop resistance to castration, that is metastatic castration-resistant prostate cancer (mCRPC), the most aggressive form of PCa with poor outcomes [3–5].

The PCa therapeutic landscape has evolved greatly in the past decade with the advent of androgen receptor pathway inhibitors (ARPIs), notably abiraterone, enzalutamide and more recently, apalutamide and darolutamide [6–9]. Although these therapies offer improved outcomes, the cancer often develops treatment resistance [10]. Chemotherapy with either docetaxel or cabazitaxel are alternatives for patients with no significant comorbidities [11]. Recently, poly (ADP-ribose) polymerase (PARP)-inhibitors have shown potential for patients with mCRPC [12–15] and have been reimbursed in Finland. Enzalutamide received full reimbursement for mCRPC after disease progression during or post-chemotherapy in December, 2015 and for first-line (1L) treatment of mCRPC in January 2018. Similarly, abiraterone was first reimbursed for mCRPC after disease progression during or post-chemotherapy in October 2015, and for 1L-treatment of mCRPC in February 2018 in Finland. Despite these advances, existing data suggest undertreatment of men with mCRPC in real-life clinical practise [16–19].

Contemporary data on real-world patients with mCRPC and their treatment journey in the Nordic region are scarce, and no data are available in Finland, particularly after the introduction of ARPIs. While pivotal for determining the efficacy and safety of treatment, clinical trials often have strict procedures and inclusion/exclusion criteria that may not fully reflect real-world practice. The purpose of this observational study, therefore, was to shed light on the largely unknown situation of these patients by describing the demographic and disease characteristics, the development of the treatment landscape, current treatment practices, and the resulting survival outcomes for the mCRPC population in real-world clinical practice in Finland.

## Methods

### Study design, setting and population

This population-based cohort study used data on patients with PCa from two Finnish hospital data lakes, specifically the hospital districts Southwest Finland (HDSF) and Helsinki and Uusimaa (HUS), as well as two national registries from the Finnish Social Insurance Institution (SII) and Statistics Finland. The two areas were selected based on representativeness of the areas, covering approximately 40% of the Finnish population, and the quality of hospital data. Data were accessed via the Auria data service and the Finnish Health and Social Data Permit Authority, Findata. Findata coordinated data collection and linkage. Data on inpatient and outpatient diagnoses, hospital medications, treatments, laboratory and pathology results were collected from patient electronic medical records (EMRs). The SII provided data on reimbursed drug purchases, and Statistics Finland provided the date and cause of death for each patient.

The study start was set at 01 January 2013 since the data lakes contained full coverage of data on hospital medications from 2013 onwards. The mCRPC cohort was identified from the whole PCa population (ICD-10 code: C61) by finding metastatic, castration-treated patients from EMRs who became castration resistant with an index date during 2013–2021. The index date was defined as that when criteria for both castration resistance and metastatic PCa were fulfilled. Patients who had been diagnosed with mCRPC before 2013 (*n* = 766) and those residing beyond the HDSF and HUS regions (*n* = 332) were excluded. Cohort formation, including detailed inclusion/exclusion criteria, is described comprehensively in Supplemental Figure 1.

### Statistical analysis and data presentation

Patient and clinical characteristics included descriptive statistics regarding the mean, median, interquartile range (IQR) and standard deviation (SD) for continuous variables, and the number of patients (N) and proportions (%) for categorical variables. Variables were described ‘as is’ with no imputation of missing data. The proportion of missing values was reported where feasible. Overall survival (OS) and treatment duration were analysed using Kaplan-Meier (KM) estimates and represented as KM curves. OS was assessed from index (mCRPC diagnosis) to death or end of follow-up (censoring event). All results were stratified based on the year of initiation of 1L-treatment (2013–2017 vs. 2018–2021) with follow-up until the end of 2021 in both time windows. All statistical analyses were performed using R (version 4.0.3).

Treatment status was determined using EMR data for records of drug administrations and prescriptions. Treatment lines were defined and analysed based on all available drug administrations and reimbursed purchases for abiraterone, cabazitaxel, docetaxel, and enzalutamide, and prescriptions for Radium-223. For each treatment type (per ATC code), single administrations, prescriptions and purchases were merged into treatment lines. Next treatment line initiation was defined as the date of the first record for the subsequent treatment type. Treatment lines were illustrated in Sankey diagrams. Cancer treatments administered and purchased before the index date were analysed for patients who had been diagnosed originally with PCa in 2013 or later, as full coverage of treatment data was only available from 2013.

## Results

**Figure 1:**
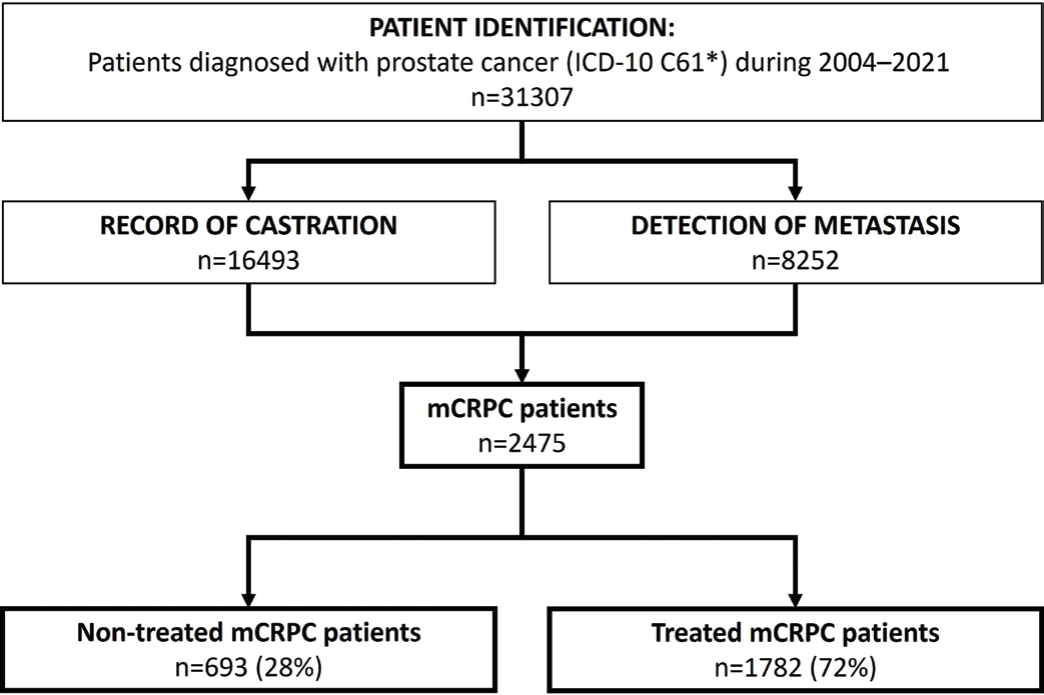
Patient identification and formation of the metastatic castration-resistant prostate cancer (mCRPC) cohort were based on records of castration, metastasis and resistance to castration in patients with prostate cancer. The final cohort was divided into treated and non-treated patients with mCRPC.

A total of 31,307 patients with PCa were identified between 01 January 2004 and 31 December 2021, and those who progressed to mCRPC between 01 January 2013 and 31 December 2021 were identified for the final study cohort that comprised 2,475 patients with mCRPC (Figure 1). In the mCRPC cohort, 759 (31%) patients were from HDSF and 1,716 (69%) from HUS (Supplementary Figure 1). The median age of patients in the total cohort identified with mCRPC was 76 (IQR: 70–82) years, with a median body-mass index of 26 (IQR: 24–30) (Table 1).

**Table 1:** Patient characteristics of all patients with metastatic castration-resistant prostate cancer (mCRPC) (whole cohort) and by non-treated and treated patients.

Variable		Whole cohort	Non-treated	Treated	Missing
N (%)		2,475	693 (28)	1,782 (72)	
Age, median (IQR)		76 (70–82)	82 (76–87)	74 (68–80)	0%
Body-mass index, median (IQR)*		26 (24–30)	26 (23–28)	27 (24–30)	31.5%
Time from PCa to mCRPC (months), median (IQR)		41 (15–97)	59 (19–107)	34 (14–90)	0
Time from castration to mCRPC (months), median (IQR)		24 (11–59)	32 (10–76)	21 (11–51)	0
PSA at mCRPC (ng/mL), median (IQR)^#^		18 (6–61)	27 (7–126)	16 (5–46)	1.3%
Charlson Comorbidity Index, *n* (%)^§^	0	1,748 (71)	400 (58)	1,348 (76)	0%
	1	549 (22)	209 (30)	340 (19)	
	2+	178 (7)	84 (12)	94 (5)	
Alive at end of follow-up		898 (36)	150 (22)	748 (42)	0%
Cause of death, *n* (%)^†^	PCa (C61)	1,109 (84)	369 (75)	740 (90)	16%
	Other	212 (16)	126 (25)	86 (10)	

* Body-mass index was determined within 2 years before and 2 months after index;

# PSA levels were determined ± 3 months from index;

§ comorbidities were determined during the 3 years before index;

† causes of death were available only until the end of 2020 so patients who died during 2021were excluded.

IQR: interquartile range; PCa: prostate cancer; PSA: prostate-specific antigen.

Patients with mCRPC were then divided into those who had been treated with life-prolonging therapies (i.e. enzalutamide, abiraterone, docetaxel, cabazitaxel or Radium-223) and referred to as ‘treated’ (72%), and those who had not (‘non-treated’, 28%). In general, non-treated patients were older (82 [IQR: 76–87] vs. 74 [IQR: 68–80] years, respectively) and had more comorbidities (Charlson Comorbidity Index [CCI] ≥ 1 in 42% vs. 24%, respectively) than treated patients. The median time from initial PCa to mCRPC diagnosis was 41 months overall (IQR: 15–97), 59 months (IQR: 19–107) for non-treated and 34 months (IQR: 14–90) for treated patients, with a large variation overall. Non-treated patients were more likely to die of causes other than PCa (Table 1).

Treated patients were further divided into two subgroups by the year of their 1L-treatment initiation (2013–2017 and 2018–2021) to elucidate changes in treatment patterns after the ARPIs were reimbursed for 1L therapy. Patient characteristics by time period are presented in Table 2. During 2013–2017, 61% (*n* = 681) of patients received life-prolonging treatments, an average of 136 treated patients per year. During 2018–2021 that increased to 80% (*n* = 1,032, an average of 258 treated patients per year). In the latter period, patients who were older and those with more comorbidities were able to receive life-prolonging treatment (*p* < 0.001 and *p* = 0.006 for age and CCI, respectively, for patients between the time periods). Ninety percent of treated patients with mCRPC died of PCa independent of the time period (Table 1).

**Table 2:** Patient characteristics treated patients subdivided by index year.

Variable		1L-treatment started		*p*
		2013–2017	2018–2021	
N (%)		681 (40)	1032 (60)	
Age, median (IQR)		72 (66–77)	75 (70–81)	< 0.001
Body-mass index, median (IQR)*		27 (25–30)	26 (24–29)	< 0.001
Time from PCa to mCRPC (months), median (IQR)		31 (14–81)	36 (15–95)	0.078
Time from castration to mCRPC (months), median (IQR)		19 (11–46)	24 (11–54)	0.014
PSA at mCRPC (ng/mL), median (IQR)^#^		20 (7–60)	13 (5–40)	< 0.001
Charlson Comorbidity Index, *n* (%)^§^	0	543 (80)	756 (73)	0.006
	1	112 (16)	213 (21)	
	2+	26 (4)	63 (6)	
Alive at end of follow-up		85 (12)	629 (61)	
Cause of death, *n* (%)^†^	PCa (C61)	497 (89)	214 (90)	
	Other	61 (11)	24 (10)	

* Body-mass index was determined within 2 years before and 2 months after index;

# PSA levels were determined ± 3 months from index;

§ comorbidities were determined during the 3 years before index;

† causes of death were available only until the end of 2020 so patients who died during 2021were excluded.

IQR: interquartile range; PCa: prostate cancer; PSA: prostate-specific antigen; mCRPC: Metastatic castration-resistant prostate cancer.

Cancer treatments administered before the index date were also analysed in patients diagnosed originally with PCa in 2013 (*n* = 1,318). Before these patients had progressed to mCRPC, their treatment included castration (99%), antiandrogens (38%), radiotherapy (35%), prostatectomy (8%) and docetaxel (21%) (Supplementary Table 1). The median time from mCRPC to treatment initiation was 43 days (95% CI: 32–50) during 2013–2017 and one day (95% CI: 1–6) during 2018–2021.

**Figure 2:**
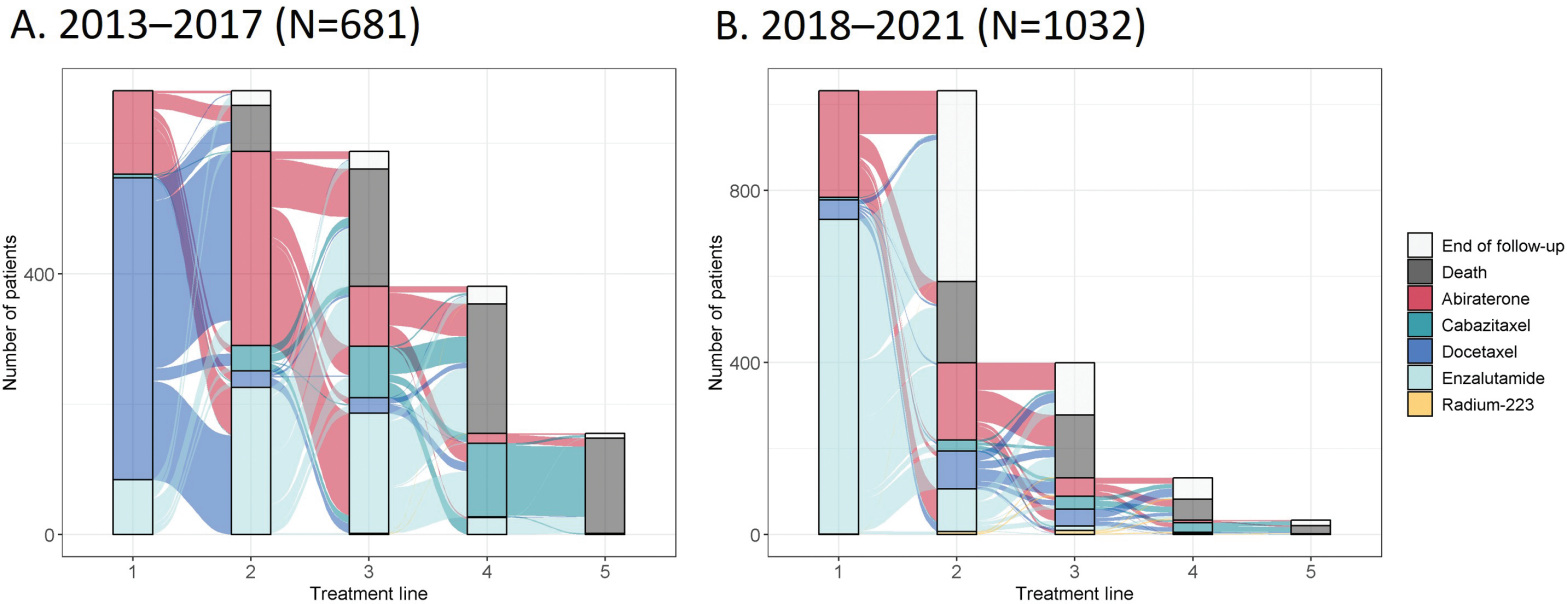
Sankey diagrams showing the metastatic castration-resistant prostate cancer (mCRPC) treatment lines for patients with 1L-treatment start between (A) 2013–2017 or (B) 2018–2021. Patients from both time periods were followed until the end of 2021.

The treatment lines are illustrated in Sankey diagrams for 2013–2017 (Figure 2A) and 2018–2021 (Figure 2B). Of the treated patients, 68% received docetaxel, 19% abiraterone or 12% enzalutamide as 1L-treatment during 2013–2017. In contrast, only 4% of patients received 1L-docetaxel, whereas 71% received enzalutamide and 24% received abiraterone 1L during 2018–2021. Only a few patients (*n* < 5) were treated with Radium-223 1L during 2013–2017 and 21 patients between 2018 and 2021. Patient numbers for each treatment type and treatment line are shown in Supplementary Table 2. The average treatment duration for 1L-treatment showed an increasing trend over time, as shown in Supplementary Figure 2.

**Figure 3:**
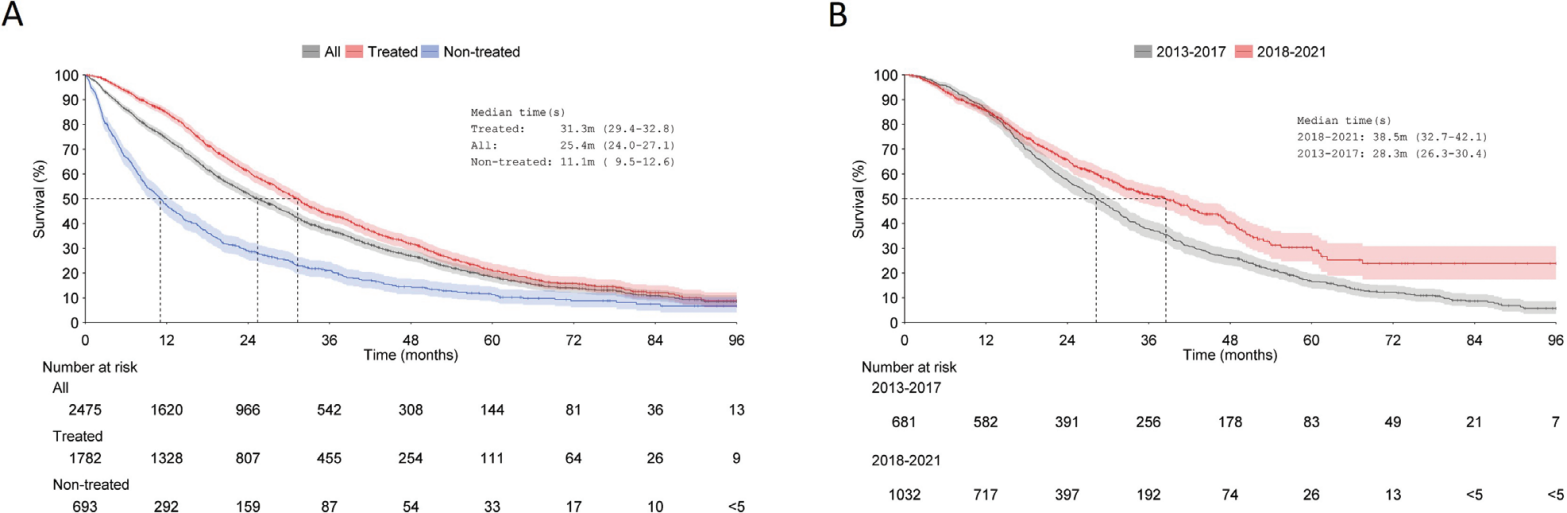
Overall survival from index to death or end of follow-up (censoring event) in (A) the whole cohort, treated patients and non-treated patients; and, (B) treated patients divided into two time periods (2013–2017 and 2018–2021) based on 1L-treatment initiation. Shaded areas represent 95% confidence intervals.

The median OS for the entire cohort was 25 months (95% CI: 24–27), 11 months for non-treated patients (95% CI: 10–13) and 31 months (95% CI: 29–33) for treated patients (Figure 3A). Post ARPI reimbursement, the median OS of treated patients increased from 28 months (95% CI: 26–30) to 39 months (95% CI: 33–42) (Figure 3B).

## Discussion

To the best of our knowledge, this is the first, real-world study of the characteristics, treatment patterns and survival outcomes of men with mCRPC in Finland. Treatment patterns changed rapidly after changes in the reimbursement status of ARPIs, favouring enzalutamide over docetaxel use. In response, the proportion of men eligible for 1L-treatment increased from 61% during the 2013–2017 period to 80% during the 2018–2021 period, and a median 10-month increase in OS was observed among treated patients.

Overall, 28% of this study cohort of men with mCRPC received no life-prolonging treatment, and likely received the best supportive care. However, this ‘non-treated’ group also may have included patients whose disease had progressed late in the study (e.g. during 2021) and who might eventually receive treatment later in the course of their disease. Non-treated patients were older, more likely to die from causes other than PCa, and had more comorbidities than treated patients, indicating a possible lack of fitness for treatment. On the other hand, the time to disease progression (castration and castration resistance) was considerably longer in the non-treated group, suggesting a less aggressive disease that did not warrant treatment. In the early study period (2013–2017), enzalutamide and abiraterone were reimbursed for disease progression during or after docetaxel-based chemotherapy, and reimbursement for 1L-treatment in patients with mCRPC was granted in 2018. Presumably, men who were more likely to tolerate chemotherapy were treated, which might also explain why the proportion of non-treated patients was higher in the early cohort (39%) compared to the cohort treated after 2018 (20%). The granting of reimbursement for abiraterone and enzalutamide in 2018 increased the number of eligible candidates for 1L-treatment in mCRPC likely due to their advantageous safety profiles compared to docetaxel. Moreover, the time from mCRPC to treatment initiation shortened from 43 days during 2013–2017 to 1 day during 2018–2021, probably due to easier administration of ARPIs. In general, treatment of mCRPC has evolved significantly with the introduction of ARPIs, and the proportion of patients using 1L-enzalutamide in our study is higher than has been shown in some other countries [20–22].

Compared to randomised clinical trials on ARPIs as 1L-treatment in mCRPC, the OS observed here was similar. Specifically, OS was 34.7 months for abiraterone [23] and 32.4 months for enzalutamide [7] in clinical trials, compared to 31 months in our study (Figure 3). However, patients in our study were generally older, with a median age of 74 years, compared to 71 and 72 years in the clinical trials [7, 23].

Recently, Freedland et al. reported real-world treatment patterns and survival for mCRPC in the fee-for-service, Medicare population in the US [16]. Despite a comparable mean age (76 years) and a similar proportion of men receiving 1L-treatment (72%) in our study compared with Freedland et al. (78%) [16], the latter observed a shorter, 23.4-month median survival after treatment initiation for mCRPC. Likewise, another study representing more than 2 million active US patients with cancer reported a shorter, 23.7-month median survival after treatment initiation [12] compared to 31.3 months in our study. The observed differences in survival between the US studies [12, 16] and ours may reflect differences in the US and Finnish populations as regards access to care (fee-for-service vs. no-fee-for-service, respectively); differences in the time periods used for cohort selection (2014–2019 vs. 2013–2021, respectively); differences in access to life-prolonging treatments (e.g. Sipuleucil-T is not available in Finland); racial differences (75% vs. 100% non-Hispanic White, respectively); and, predominantly community-based, oncology practices in US versus academic centres in our study.

As regards the Prostate Cancer Registry study of 3,003 patients with mCRPC from registries in 16 countries, Chowdhury et al. [24] have presented data on the real-world effectiveness of 1L-treatment with abiraterone, enzalutamide and docetaxel in mCRPC, while Bjartell et al. [25] have analysed treatment sequences and outcomes (progression and survival) for these treatments. OS in these studies for patients enrolled during 2013–2016 was 27–28 months, depending on treatment, which is similar to the OS of 28.3 months during 2013–2017 that we report here. Moreover, a Swedish study in which treatment patterns in 1,699 patients diagnosed between 2006 and 2015 with castration-resistant prostate cancer (CRPC) were analysed, 463 patients had newly diagnosed mCRPC [19]. Only 50% of patients received life-prolonging treatment during 2013–2015, and the median OS from CRPC diagnosis during 2006–2015 was only 20 months (IQR 8.4–45), which may reflect the early study period during which most patients in the Stockholm cohort were treated with docetaxel as 1L-treatment [14].

Our study required complex algorithms due to limitations in data entry practices as regards disease progression in routine clinical care. Since mCRPC status is not systematically recorded in patient charts or registries, here it was deduced and confirmed *post hoc* using different variables and selection criteria (see Supplemental Figure 1). The complex cohort formation process and uncertainties in data entry practices may have led to some patients with mCRPC not being included in our cohort; however, the strict selection criteria ensured that most of the cohort were considered to be true mCRPC patients. Dividing patients into two time windows and following both cohorts until the end of 2021 means that the data for patients from the earlier period are more mature. Finally, since treatment can be initiated sometimes even years after index (mCRPC), there was a risk for an immortal time bias in the treated group. The 43-day median time to treatment initiation may have caused a small bias towards a longer OS for treated patients in the 2013–2017 subgroup, an effect that could be neglected for 2018–2021 as the median time was only one day.

Finnish hospital data lakes and the possibility to link deep, clinical data with national registry data, combined with equal access, non-fee-for-service universal healthcare, represent an efficient opportunity for real-world studies, and represent a strength of this study. Additionally, the study cohort comprised patients from two large hospital districts that cover approximately 40% of the Finnish population. These findings, therefore, are largely applicable to the rest of Finland due to uniform, national treatment practices for PCa. We would also expect good generalisability of our findings to other Nordic countries based on similar healthcare systems, although this would be more challenging beyond the Nordic region due to differences in healthcare systems and/or populations, as we have already discussed.

In conclusion, the treatment and outcomes of patients with mCRPC have changed significantly after ARPIs were granted reimbursement status in Finland. More patients with the most aggressive form of PCa are now able to receive life-prolonging treatment leading to better OS in the real-world setting.

## Supplementary Material

Real-world treatment patterns and survival outcomes in men with metastatic castration-resistant prostate cancer in Finland: a national, population-based cohort study

## Data Availability

Since this study is based on the secondary use of healthcare registry data, these data cannot be shared openly.
