# Supplementary material for: Real-world treatment patterns and survival outcomes in men with metastatic castration-resistant prostate cancer in Finland: a national, population-based cohort study
Source: Acta Oncol. 2025 Jan 29;64:42173. doi: 10.2340/1651-226X.2025.42173 (PMC11808809; doi:10.2340/1651-226X.2025.42173)

Supplementary material has been published as submitted. It has not been copyedited, or typeset by Acta Oncologica

Supplementary Material

Methods

Supplementary Figure 1. Detailed cohort formation.

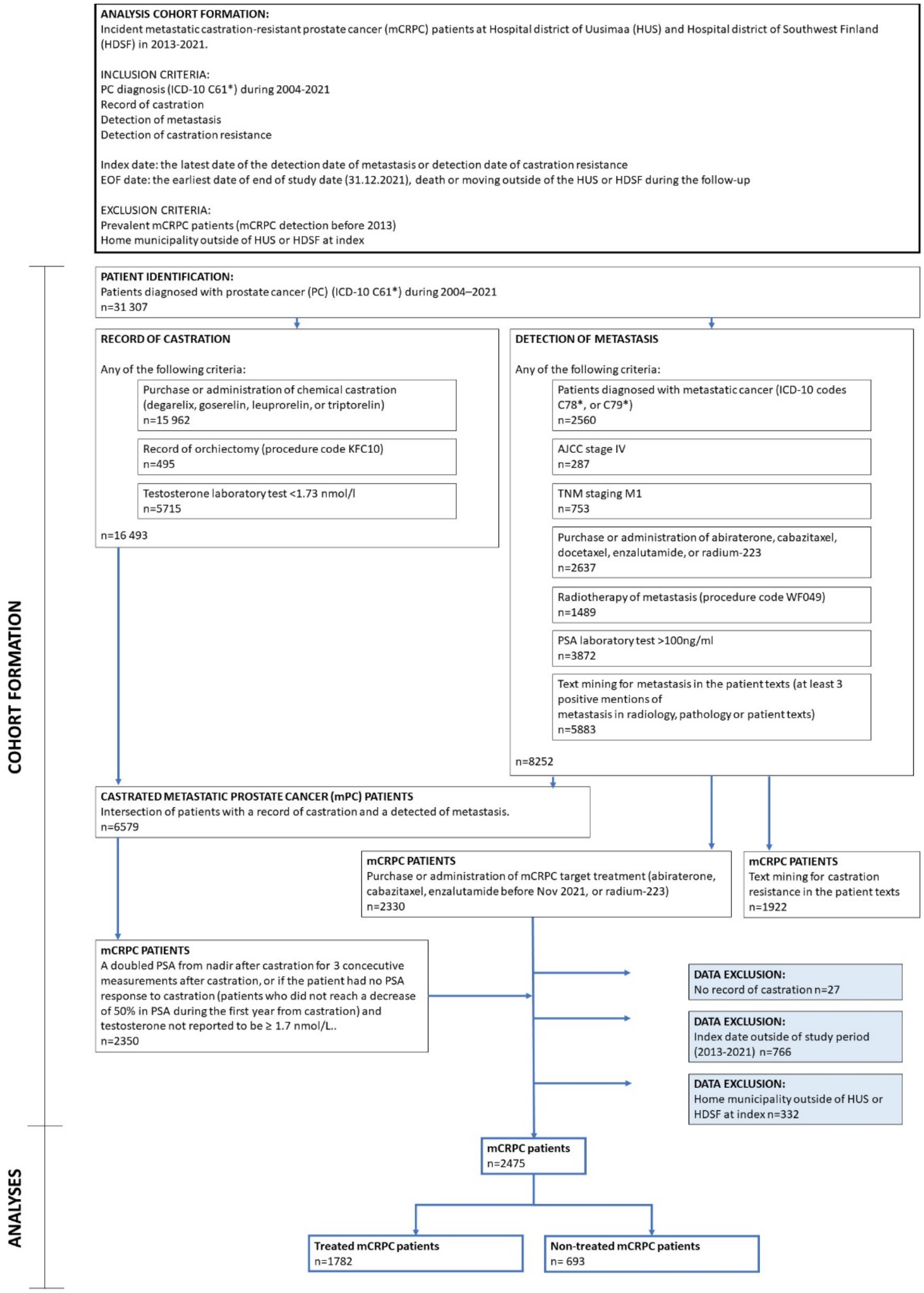

RESULTS

Supplementary Table 1. Treatments used after diagnosis of prostate cancer, but before diagnosis of metastatic castration-resistant prostate cancer for patients diagnosed in 2013 or later.

	**Whole cohort**		**Treated**		**Non-treated**	
**Treatment**	**N**	**%**	**N**	**%**	**N**	**%**
Castration (chemical or surgical)	1305	99	1019	99	286	98
Bilateral orchiectomy	70	5	37	4	33	11
Chemical castration (degarelix, leuprorelin, triptorelin, goserelin)	1268	96	1003	98	265	90
Antiandrogens (flutamide, bicalutamide)	502	38	403	39	99	44
Any radiotherapy	460	35	396	39	64	22
Radical radiotherapy	214	16	193	19	21	7
Radiotherapy of metastasis	156	12	141	14	15	5
Palliative radiotherapy	128	10	115	11	13	4
Adjuvant radiotherapy	89	7	69	7	20	7
Prophylactic radiotherapy	47	4	43	4	0	0
Radiotherapy of local residual tumour	8	1	6	1	0	0
Total excision of prostate and seminal vesicles	108	8	90	9	18	6
Docetaxel	279	21	275	27	<5	
Zoledronic acid	17	1	17	2	0	0
Denosumab	12	1	12	1	0	0

Supplementary Figure 2. Kaplan-Meier curves of treatment duration from start to end of treatment with enzalutamide, abiraterone, docetaxel, and cabazitaxel for first-line treatment of metastatic castration-resistant prostate cancer for patients starting 1L treatment during 2013-2017 (A) or 2017-2021 (B). Due to the low number of patients treated with cabazitaxel, docetaxel and cabazitaxel were combined to one group. Shaded areas represent 95% confidence intervals.

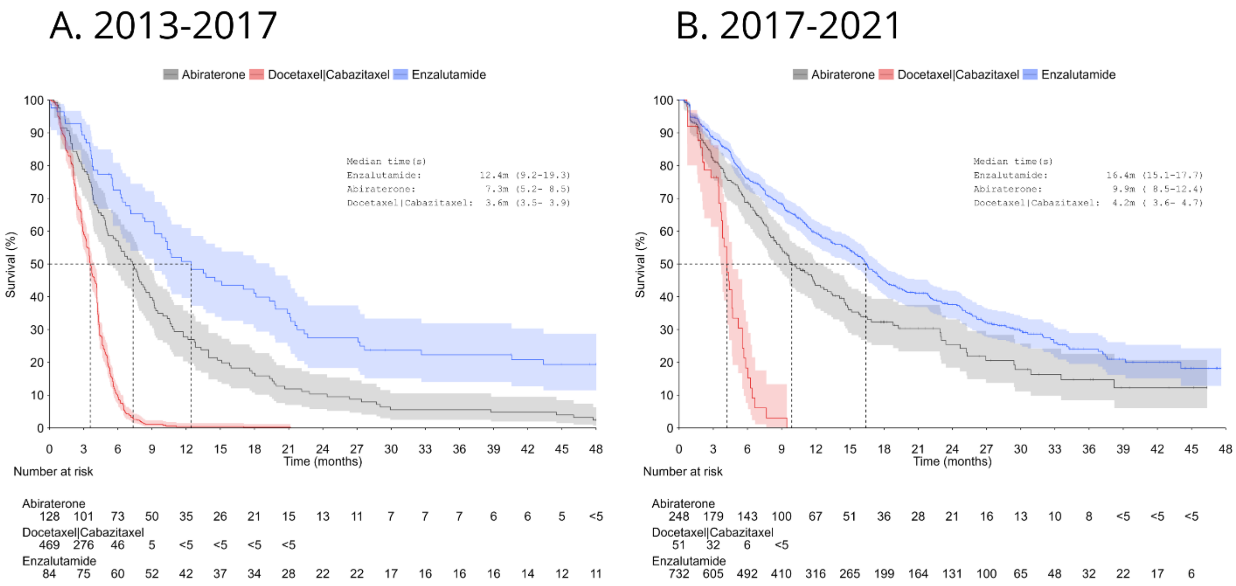

Supplement: Real-world treatment patterns and survival outcomes in men with metastatic castration-resistant prostate cancer in Finland: a national, population-based cohort study [file AO-64-42173-s1.pdf]
